# Quantum-well states at the surface of a heavy-fermion superconductor

**DOI:** 10.1038/s41586-023-05830-1

**Published:** 2023-03-22

**Authors:** Edwin Herrera, Isabel Guillamón, Víctor Barrena, William J. Herrera, Jose Augusto Galvis, Alfredo Levy Yeyati, Ján Rusz, Peter M. Oppeneer, Georg Knebel, Jean Pascal Brison, Jacques Flouquet, Dai Aoki, Hermann Suderow

**Affiliations:** 1 Facultad de Ingeniería y Ciencias Básicas, Universidad Central, Bogotá, Colombia; 2 Departamento de Física, Universidad Nacional de Colombia, Bogotá, Colombia; 3 Laboratorio de Bajas Temperaturas y Altos Campos Magnéticos, Unidad Asociada UAM/CSIC, Departamento de Física de la Materia Condensada, Instituto Nicolás Cabrera and Condensed Matter Physics Center (IFIMAC), Universidad Autónoma de Madrid, Madrid, Spain; 4 School of Engineering, Science and Technology, Universidad del Rosario, Bogotá, Colombia; 5 Departamento de Física Teórica de la Materia Condensada, Instituto Nicolás Cabrera and Condensed Matter Physics Center (IFIMAC), Universidad Autónoma de Madrid, Madrid, Spain; 6 Department of Physics and Astronomy, Uppsala University, Uppsala, Sweden; 7 University Grenoble Alpes, CEA, Grenoble-INP, IRIG, PHELIQS, Grenoble, France; 8 Institute for Materials Research (IMR), Tohoku University, Oarai, Japan

**Keywords:** Superconducting properties and materials, Electronic properties and materials, Surfaces, interfaces and thin films

## Abstract

Two-dimensional electronic states at surfaces are often observed in simple wide-band metals such as Cu or Ag (refs. ^1–4^). Confinement by closed geometries at the nanometre scale, such as surface terraces, leads to quantized energy levels formed from the surface band, in stark contrast to the continuous energy dependence of bulk electron bands^2,5–10^. Their energy-level separation is typically hundreds of meV (refs. ^3,6,11^). In a distinct class of materials, strong electronic correlations lead to so-called heavy fermions with a strongly reduced bandwidth and exotic bulk ground states^12,13^. Quantum-well states in two-dimensional heavy fermions (2DHFs) remain, however, notoriously difficult to observe because of their tiny energy separation. Here we use millikelvin scanning tunnelling microscopy (STM) to study atomically flat terraces on U-terminated surfaces of the heavy-fermion superconductor URu_2_Si_2_, which exhibits a mysterious hidden-order (HO) state below 17.5 K (ref. ^14^). We observe 2DHFs made of 5f electrons with an effective mass 17 times the free electron mass. The 2DHFs form quantized states separated by a fraction of a meV and their level width is set by the interaction with correlated bulk states. Edge states on steps between terraces appear along one of the two in-plane directions, suggesting electronic symmetry breaking at the surface. Our results propose a new route to realize quantum-well states in strongly correlated quantum materials and to explore how these connect to the electronic environment.

## Abstract (web summary)

By using millikelvin scanning tunnelling microscopy to study atomically flat terraces on U-terminated surfaces of the heavy-fermion superconductor URu_2_Si_2_, the two-dimensional heavy fermions are shown to form quantum-well states on the surface.

## Main

Heavy fermions form a unique class of quantum materials that exhibit exceptional properties related to their narrow electronic-band dispersion^12^. Previous experiments on heavy fermions showed that reducing the dimensionality leads to enhanced electronic correlations and strong coupling superconductivity^15,16^. Furthermore, narrow surface bands with a Dirac dispersion were found in a semiconducting heavy fermion^17^. In spite of intensive investigations, 2DHFs have not been observed at surfaces of superconducting compounds and no quantum-well states owing to lateral confinement of heavy electron states have been realized.

**Fig. 1:**
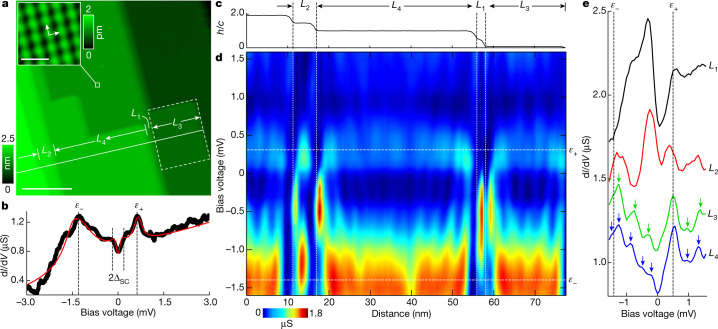
Tunnelling conductance on URu_2_Si_2_ terraces. **a**, STM topography image of several U-terminated terraces on URu_2_Si_2_, cleaved perpendicular to the *c* axis. The distance between terraces is *c*/2, that is, half a unit cell. The inset shows a zoom into the region at the white square and reveals the square U atomic surface lattice (arrows indicate the in-plane crystalline directions). The dashed rectangle represents the field of view on which we focus in Fig. 2. More details about the surface termination is provided in Methods and in Extended Data Fig. 1. Scale bars, 20 nm (main), 1 nm (inset). **b**, Tunnelling conductance averaged along the white line in **a**. The dashed lines provide the features at *ε*_−_, *ε*_+_ and the superconducting gap discussed in the Methods. **c**, Height profile normalized to the *c*-axis lattice constant along the white line in **a**, indicating terraces *L*_1_ to *L*_4_. **d**, Tunnelling conductance versus distance along the white line in **a**. **e**, Tunnelling conductance curves at different terraces. The arrows mark the positions of peaks arising from quantization due to lateral confinement. Data taken at 0.1 K.

**Extended Data Fig. 1:**
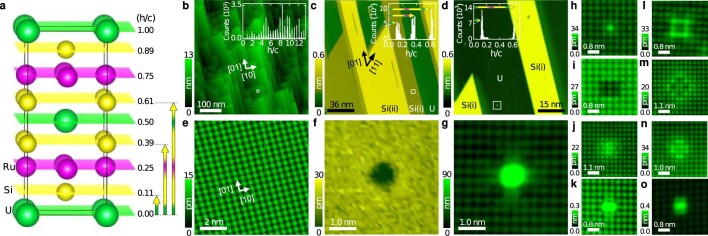
Different surface terminations in URu_2_Si_2_. **a**, URu_2_Si_2_ crystal structure. U atoms are shown in green, Ru in magenta and Si in yellow. We show using the same colours the corresponding planes and indicate the distances between planes, normalized to the *c*-axis lattice parameter. With coloured arrows, we highlight the distance between the planes observed in the STM images. **b**, STM topography image at a surface with terraces. As we discuss in the text, these are all U-terminated terraces. The height scale is given by the bar on the left. In the upper-right inset, we show a height histogram (distances normalized to the *c*-axis lattice constant). Notice that all peaks are located at an integer of *c*/2. The crystal axes are shown as white arrows. The small white square is the area shown in **e**. **c**,**d**, STM topography images at surfaces showing terraces with a triangular shape. These are distinct terraces. The crystal axes are shown as arrows. In the upper-right inset, we show the height histogram, with distances between terraces having different sizes. Coloured arrows are as in **a** and identify height differences between the U and Si terraces. The small white squares in **c** and **d** provide the areas shown in **f** and **g**, respectively. We show other kinds of defect in **h** to **o**, with the colour scale given by the bars on the left of each image. All data were taken at 100 mK with a tunnelling current *I*_tunnel_ = 10 nA and a bias voltage *V*_Bias_ = 10 mV.

**Extended Data Fig. 2:**
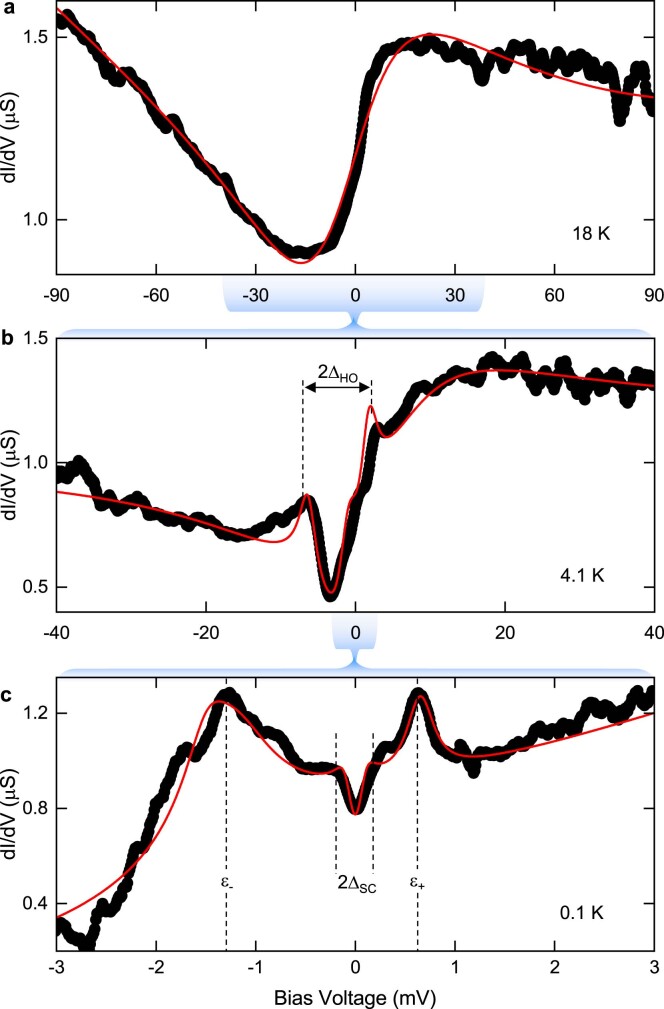
Tunnelling conductance of the HO state. **a**, Tunnelling conductance versus bias voltage at a temperature above the HO transition temperature (17.5 K) is shown as black points. The red line is a fit based on the Fano lineshape owing to parallel tunnelling paths into light and heavy bands. **b**, Tunnelling conductance inside the HO state versus bias voltage is shown as black points. Notice the reduction of the bias voltage range, to highlight the features associated to HO within the Fano lineshape. The HO gap is schematically marked by an arrow. The red line is a fit as described in the text. **c**, At the lowest temperatures and focusing on very low bias voltages, we observe the tunnelling conductance represented as black points. The red line is a fit with a model described in Methods. We show by dashed lines the features at *ε*_+_, *ε*_−_ and at the superconducting gap value Δ_SC_. Temperatures at which the data were taken are provided in each panel.

We investigate the heavy-fermion superconductor URu_2_Si_2_, which exhibits correlated narrow electron bands crossing the Fermi level and undergoes a transition to the HO phase characterized by an as yet unknown order parameter^14^. A partial gap opens in the electronic band structure below *T*_HO_ = 17.5 K, out of which an unconventional superconducting state develops below *T*_c_ = 1.5 K (refs. ^14,18^). The surface electronic states observed until now mostly have small effective masses^19–22^. Using STM to investigate small-sized, atomically flat terraces on the U-terminated surface of URu_2_Si_2_ at sub-kelvin temperatures, we observe clearly 2DHFs with an effective mass 17 times the free electron mass, as well as quantization owing to lateral confinement and uncover the surface–bulk interaction. In Fig. 1a, we present a STM image of terraces of U-terminated surfaces on URu_2_Si_2_ (see Extended Data Fig. 1 for the surface termination). Our starting point is the tunnelling conductance obtained in a small range of a few mV around the Fermi level and at a temperature of 0.1 K, well below the superconducting critical temperature, shown in Fig. 1b (see Extended Data Fig. 2 for a larger bias voltage range).

## Quantum-well states by confinement

We focus on the tunnelling conductance along the white line shown in Fig. 1a. Along this line, we identify four terraces of different sizes, *L*_1_ ≈ 2 nm, *L*_2_ ≈ 5.5 nm, *L*_3_ ≈ 20 nm and *L*_4_ ≈ 38.5 nm (Fig. 1c). We observe a strong bias voltage and position dependence of the tunnelling conductance, which is different for each terrace as illustrated in Fig. 1d. We show in Fig. 1e representative tunnelling conductance curves at each terrace, in which we identify a set of regular peaks.

**Fig. 2:**
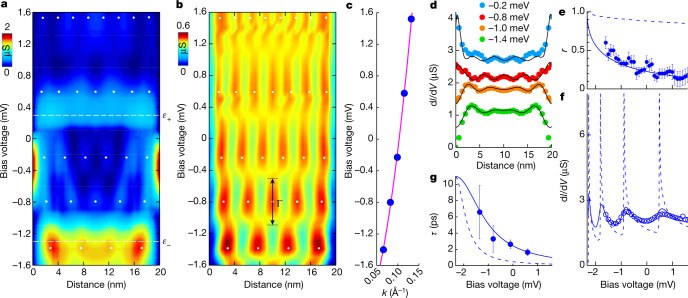
2DHFs and electron-in-a-box quantization. **a**, Tunnelling conductance at the *L*_3_ terrace is shown by a colour scale as a function of the distance (taken at 0.1 K). **b**, The same data as in **a** but with a subtracted background (see Methods and Extended Data Fig. 3). The white dots in **a** and **b** mark the position of peaks in the conductance. The black arrow shows the width of the quantized levels, Γ, described in the text. **c**, Points show the reciprocal space position of the white dots in **a** and **b**. The magenta line provides the electron dispersion relation with m* = 17*m*_0_. **d**, The lines show calculations and coloured points the measured tunnelling conductance. **e**, The dashed blue line shows the reflection coefficient *r* obtained in Cu(111) (see Methods). The continuous blue line is the calculated *r* assuming m* = 17*m*_0_. Points are the results obtained from the experiment. **f**, Tunnelling conductance as a function of the bias voltage is shown by blue circles at the centre of the terrace in **b**. The dashed blue line is for perfect reflection, *r* ≈ 1, and the continuous line for *r* ≈ 0.4. **g**, Points show the lifetime of the quantum-well states, *τ*, as a function of the bias voltage. The dashed blue line is the expectation for a two-dimensional electron gas and the continuous line describes quantum states whose width is set by their interaction with the heavy quasiparticles of the bulk.

**Extended Data Fig. 3:**
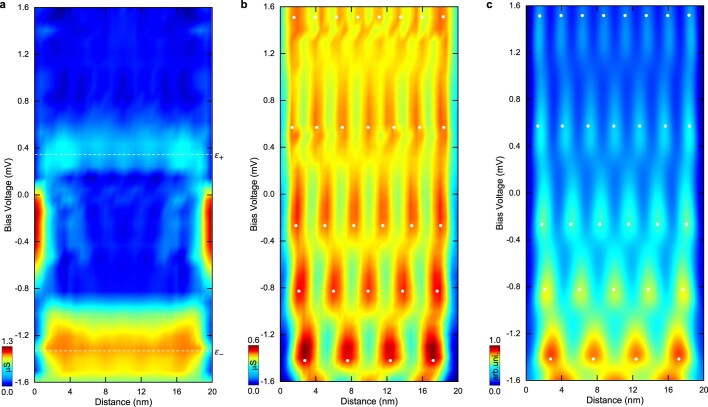
Subtracted tunnelling conductance background and quantized density of states pattern. **a**, Bias voltage dependence of the subtracted tunnelling conductance background versus distance. **b**, Bias voltage dependence of the tunnelling conductance obtained after the subtraction. **c**, Fabry–Pérot calculation using the parameters discussed in the text. Quantized levels are represented by white points. Colour scale from blue (low conductance) to red (high conductance) represents values given in the bars at the bottom left.

**Extended Data Fig. 4:**
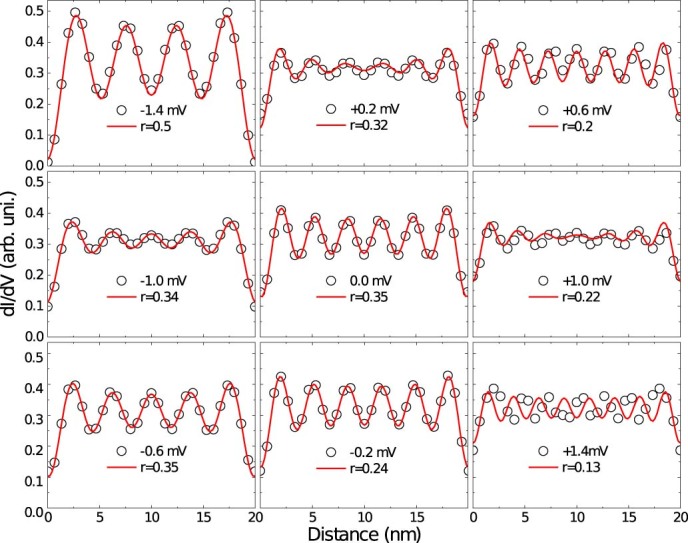
Fits of the tunnelling conductance. We show as circles the tunnelling conductance as a function of distance for the bias voltages indicated in the legend of each panel (taken at 0.1 K). Lines are fits described in Methods, with–in addition–a nearly constant background to account for the features at *ε*_−_ and *ε*_+_ and the features of the 1DES.

**Extended Data Fig. 5:**
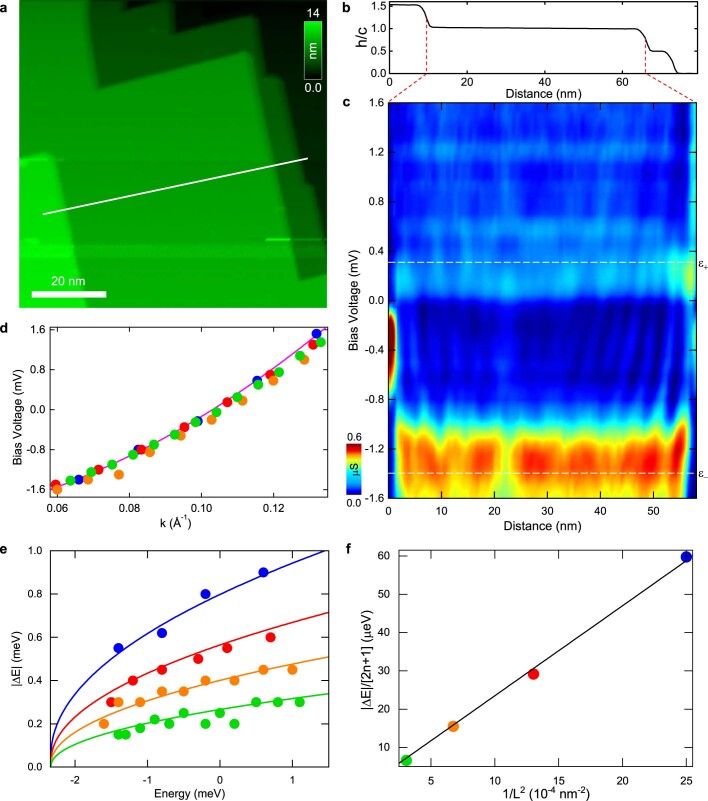
Dispersion relation and quantization on different terraces. **a**, STM image on a field of view containing a few U-terminated terraces (taken at 0.1 K). **b**, Height profile (normalized with the *c*-axis lattice constant) along the white line in **a**. **c**, Tunnelling conductance along the central terrace (*L* ≈ 57 nm) of the profile shown in **b** (colour scale from red, high conductance, to blue, low conductance). We mark the position of the features at *ε*_+_ and *ε*_−_ with dashed white lines. **d**, Dispersion relation of the 2DHF (magenta line). Points are the positions of the quantized levels obtained from different terraces as described in the text (size *L* of each terrace is 20 nm blue, 28 nm red, 38.5 nm orange and 57 nm green). **e**, separation between energy levels Δ*E* as a function of the energy (coloured points following the colour code of **d**). Lines are a square-root dependence, from $\Delta E={E}_{n+1}-{E}_{n}=\left(\frac{{\hbar }^{2}{\pi }^{2}}{2{m}^{* }{L}^{2}}\right)\left(2n+1\right)$. **f**, Average of Δ*E* (colour code as in **d**) as a function of the inverse of *L*^2^.

Let us analyse the terrace *L*_3_ (dashed rectangle in Fig. 1a). We present a symmetrized map of the tunnelling conductance in Fig. 2a (see Methods for further details). We identify a set of peaks in the tunnelling conductance, which evolve in both position and bias voltage. Subtracting the features at *ε*_−_ and *ε*_+_ (details provided in Methods and in Extended Data Figs. 3 and 4), we obtain the pattern shown in Fig. 2b, which shows the lateral quantization of 2DHFs. The quantization pattern for confined electrons resembles the Fabry–Pérot expression for an interferometer made by partially reflecting mirrors^23^ (reflection coefficient *r* and the phase shift *ϕ* are the free parameters; details in Extended Data Fig. 4 and results on different terraces in Extended Data Fig. 5). Lateral quantization results from interfering wavefunctions partially reflected at steps. Quantized levels obtained from the Fabry–Pérot expression are the white dots in Fig. 2a,b, whose position coincides well with the peaks in the conductance pattern observed in the experiment. In Fig. 2c, we plot as points the position of the peaks as a function of the wavevector *k* and as a line the dispersion relation *E* = *E*_0_ + *ħ*^2^*k*^2^/(2m*), in which m* is the effective mass and *E*_0_ the bottom of the band. We obtain *E*_0_ = −2.3 meV and m* = 17*m*_0_, with *m*_0_ the free electron mass, that is, we find that the 2DHF is derived from a massive surface electron state. A detailed comparison of the tunnelling conductance versus position with the square of wavefunctions confined by a lateral potential leads to excellent fits, shown in Fig. 2d. The phase shift *ϕ* determines the position and energy of the peaks (white dots in Fig. 2a,b) and the best account of our observations is obtained with *ϕ* = −π. We find values around *r* ≈ 0.2, which slightly increase when approaching *E*_0_ (Fig. 2e). The low value of *r* is also found in surface states of simple metals; for example, *r* is between 0.2 and 0.4 in Ag and Cu (refs. ^23–25^). However, the energy dependence (Fig. 2e) at the surface of URu_2_Si_2_ is completely different to that in usual metals. Although *r* varies mildly in Cu or Ag in the range of a few eV (see dashed line in Fig. 2e and refs. ^23–25^), here we observe instead that *r* decreases markedly in a range of a few meV (points in Fig. 2e). We can reproduce the observed dependence of *r* versus bias voltage assuming a potential well^23,24^ (continuous line in Fig. 2e; details provided in Methods and Extended Data Figs. 3 and 4). It is also insightful to trace the tunnelling conductance as a function of the bias voltage at the centre of the terrace in Fig. 2b (circles in Fig. 2f) and compare it to the expectation for *r* ≈ 1 (dashed line in Fig. 2f) and for *r* ≈ 0.4 (continuous line in Fig. 2f). We see that, for a reduced *r*, both the periodicity and shape of the tunnelling conductance are well explained in an energy range of a few meV, that is, two orders of magnitude below the energy range observed in conventional metals^1–4,23–26^.

To further investigate the quantized levels, we have fitted each peak to a Lorentzian function, whose width Γ (black arrow in Fig. 2b) provides the lifetime *τ* (Fig. 2g) of the quantum-well states. Taking a two-dimensional electron gas, we expect $$\hbar /\tau ={\Gamma }_{0}+(| {E}_{0}| /4\pi ){\left[(E+| {E}_{0}| )/{E}_{0}\right]}^{2}$$$$| {\rm{ln}}| (E+| {E}_{0}| )/{E}_{0}| -{\rm{ln}}(2{q}_{{\rm{TF}}}/{k}_{{\rm{F}}})-1/2| $$, with *ħ* the reduced Planck’s constant, *q*_TF_ = 0.0906 Å^−1^ the Thomas–Fermi screening length and *k*_F_ the Fermi wavevector^27^ (dashed line in Fig. 2g). Our data are not well reproduced by this expression. Taking instead $$\hbar /\tau ={\Gamma }_{0}+(| {E}_{0}| /4\pi ){\left[(E+| {E}_{0}| )/{E}_{0}\right]}^{2}$$ with *E*_0_ = −2.3 meV and Γ_0_ ≈ 60 μeV (continuous line in Fig. 2g), we find a much better account of our data^27,28^. The latter expression takes into account the connection between the 2DHF and bulk states, showing that, in our experiments, the lifetime *τ* is set by the decay of the 2DHF into heavy-fermion bulk states. Quantum-well states sense the bulk correlations, given by the quadratic energy term in *ħ*/*τ*. This has been observed in surface states of noble metals, monolayers of Pb and in Sb. However, in those cases, the energy range was three orders of magnitude above the one we discuss here^3,6,11,28^.

From the obtained value of Γ_0_ ≈ 60 μeV, we estimate the lifetime of the ground state as *τ*_0_(*E*_0_ = −2.3 meV) = *ħ*/Γ_0_ ≈ 11 ps. Similarly, the lifetime of states close to the Fermi level is *τ*(*E* = 0) = *ħ*/Γ(*E* = 0) ≈ 3 ps. We can also estimate a value for a mean free path, *ℓ*_0_ = *v*_F_*ħ*/Γ(0) ≈ 0.14 μm, with *v*_F_ the Fermi velocity of URu_2_Si_2_. This value is on the same order of magnitude as those observed in ultraclean URu_2_Si_2_ single crystals^29^.

**Extended Data Fig. 6:**
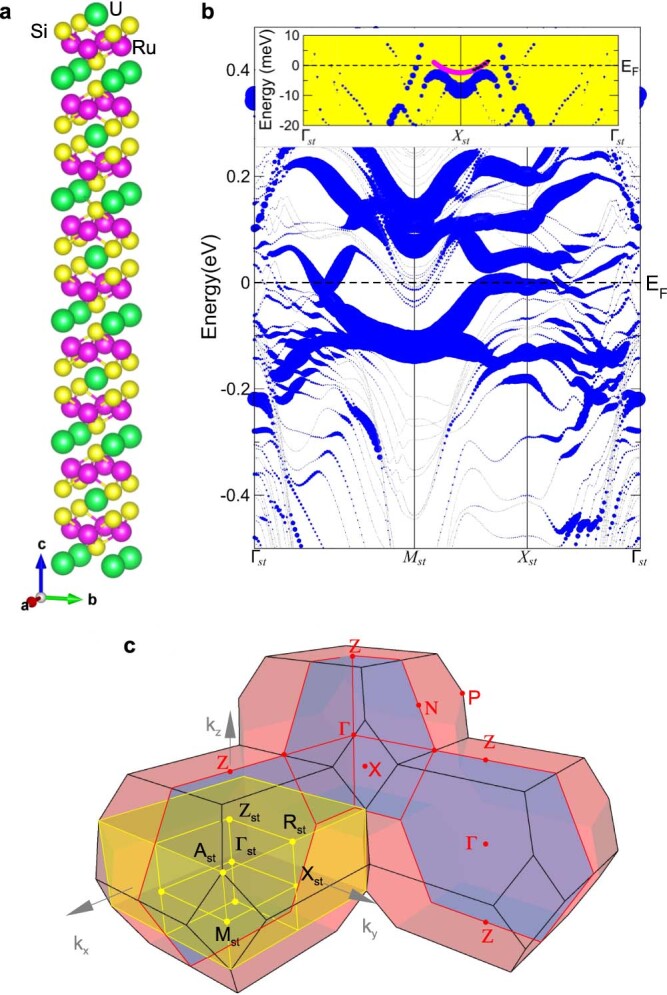
DFT calculations of the surface band structure around the *X* point of the simple tetragonal Brillouin zone. **a**, U-terminated supercell structure of URu_2_Si_2_ used for DFT calculations. **b**, Band structure of URu_2_Si_2_ in a slab calculation (blue points), described in the text, along the high-symmetry directions of the simple tetragonal Brillouin zone, Γ_st_, *M*_st_ and *X*_st_. The size of the points provides the U spin-up character of the bands. In the upper inset, we show a zoom-in around the *X*_st_ point. The magenta line provides the dispersion relation compatible with our experiments and the black points are the quantized levels we identified (from Fig. 2c). **c**, The usual Brillouin zone construction of URu_2_Si_2_, with the tetragonal Brillouin zone (red lines) and the simple tetragonal (st) construction (yellow lines) used to describe the low-temperature HO phase.

To vindicate the existence of a heavy-fermion surface state, we performed density functional theory calculations of the surface band structure of a slab of URu_2_Si_2_ (Extended Data Fig. 6). We find a shallow, U-derived f-electron band with a flat dispersion relation compatible with our experiments around the *X* point of the simple tetragonal Brillouin zone. The bulk electronic spectrum is gapped in this part of the Brillouin zone^21,22^. The rest of the Brillouin zone provides surface states with much smaller effective masses.

## One-dimensional edge states

**Fig. 3:**
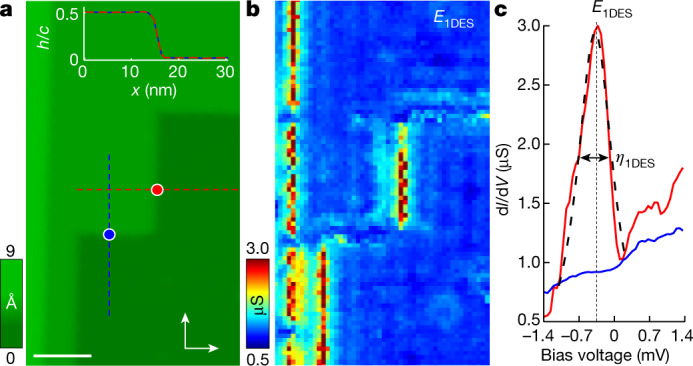
1DESs at steps. **a**, STM image of a set of U terraces separated by steps half a unit cell in size. The colour scale provides the height, following the bar on the left. In the upper inset, we show profiles, taken along the red and blue dashed lines. The white arrows indicate the two in-plane crystal directions, which are crystallographically equivalent. The red and blue circles provide the positions at which the red and blue curves in **c** were taken. Scale bar, 10 nm. **b**, Tunnelling conductance map at the energy of the 1DES, *E*_1DES_. The colour scale is shown on the left. **c**, We observe the 1DES when crossing a vertical step (dashed red line in **a**), as shown by the tunnelling conductance versus bias voltage (red line), but not along the other in-plane crystal axis (blue line). The dashed black line is a fit and the horizontal arrow marks the width of the 1DES, *η*_1DES_ (more details in Methods and Extended Data Fig. 7).

**Extended Data Fig. 7:**
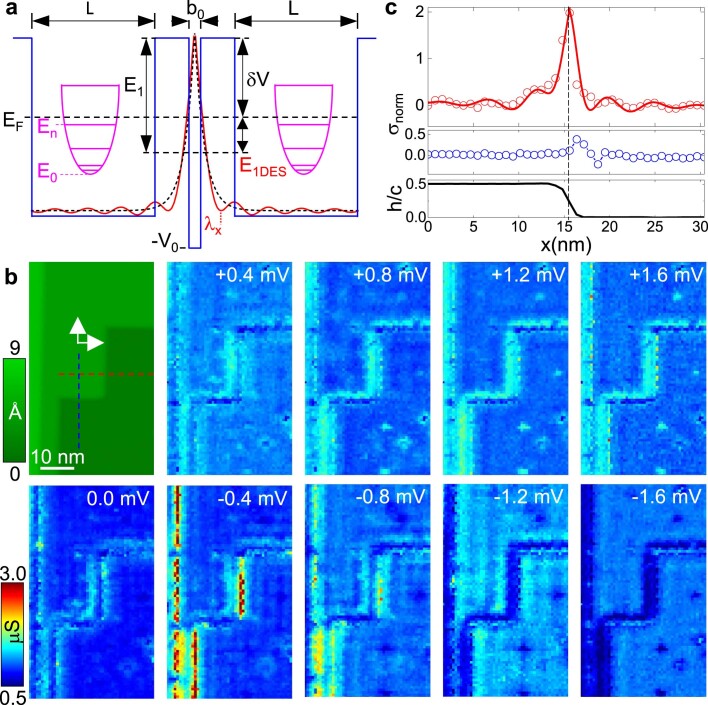
1DES and HO within U layers. **a**, Schematic representation of the parameters used to describe the 1DES at a step between two consecutive terraces of length *L*. We represent the quantized levels of the confined 2DHF on each terrace with pink colour. The dashed black (continuous red) line represents the exponential behaviour of the 1DES without (with) coupling to the quantized levels. **b**, Topography image along a step between two consecutive terraces formed by U layers is shown in the top-left panel. The colour scale is shown as a bar on the left, in Å. White arrows provide the in-plane crystalline axis. The other panels are tunnelling conductance images (colour scale provided at the left) in the same field of view for different values of the bias voltage (taken at 0.1 K). Notice the different shape of the 1DES along steps that are located on the two in-plane directions of the U atom lattice. **c**, Circles in red and blue represent the tunnelling conductance as a function of distance (referred to the tunnelling conductance far from the edge at *x* = 50 nm) at the bias voltage at which the 1DES appears *V* ≈ −0.4 mV, *σ*_norm_(−0.4 mV) = *σ*(*V* = −0.4 mV,*x*) − *σ*(*V* = −0.4 mV,*x* = 50 nm) along the red and blue lines in the top-left panel of **b**. The continuous red line is the fit of the 1DES (equation (8)) plus the quantized level model (equation (3)). The bottom panel shows (black line) the STM height profile along the red and blue lines in the top-left panel of **b** in units of the *c*-axis lattice constant.

In Fig. 3, we show that the 2DHF is peculiarly modified at the steps separating terraces at which one-dimensional edge states (1DESs) appear. 1DESs were previously observed in simple metals, in which a gap opens at the step and is filled with a very large density of states at *E*_1DES_ by the 1DES^3,8,11,24,25,30^. The width of the conductance peak at *E*_1DES_, *η*_1DES_, results from inelastic scattering into bulk states^24,25^. Here we find that the features in the tunnelling conductance completely change at a step (Fig. 3a,b). We find a high peak at *E*_1DES_ ≈ −0.38 meV (Fig. 3b,c) in steps, but notably only along one of the two equivalent in-plane axes, that is, an in-plane symmetry breaking occurs. The HO is known to cause breaking of the body-centred translation symmetry^14^, leading to inequivalent electronic properties in subsequent U layers, which is consistent with our measurements (details in Methods and Extended Data Fig. 7). This should reduce inelastic scattering and favour the formation of a 1DES. Here, however, we observe a rather unique situation in which the edge state is either observed or not, on two crystallographically equivalent in-plane axes. This indicates spontaneous symmetry breaking of the fourfold rotational symmetry close to the surface. Such in-plane symmetry breaking has been proposed^31^ for bulk URu_2_Si_2_ but has been difficult to detect. This symmetry breaking would cause an orthorhombicity given by a tiny difference in the basal-plane lattice constants *a* and *b* ($\frac{| \,a-b\,| }{(a+b)}\approx 1{0}^{-5}$)^32^. Other experiments could, however, not confirm such in-plane symmetry breaking^33^, which could be favoured by defects. Recent group theory considerations and nuclear magnetic resonance (NMR) data propose that the HO state can belong to four space groups, #126, #128, #134 and #136, all having the same crystal structure as the high-temperature state^34^. NMR experiments suggest the presence of fourfold symmetry at Ru, Si and U sites, narrowing down the most probable choice to #126 (refs. ^34,35^). Notably, although these measurements gave absence of fourfold symmetry breaking in bulk URu_2_Si_2_, our STM data sensitive to individual uranium layers clearly show an in-plane symmetry breaking in the 1DES. We note that a possible source of changes in the electronic structure that could also affect the 1DES is modifications of the valence of U edge atoms^21^. Nonetheless, the symmetry-breaking in the 1DES suggests a deeper origin, which pinpoints that the 1DES serves as a sensitive probe of fundamental electronic properties of the near-surface U lattice.

## Superconductivity

**Fig. 4:**
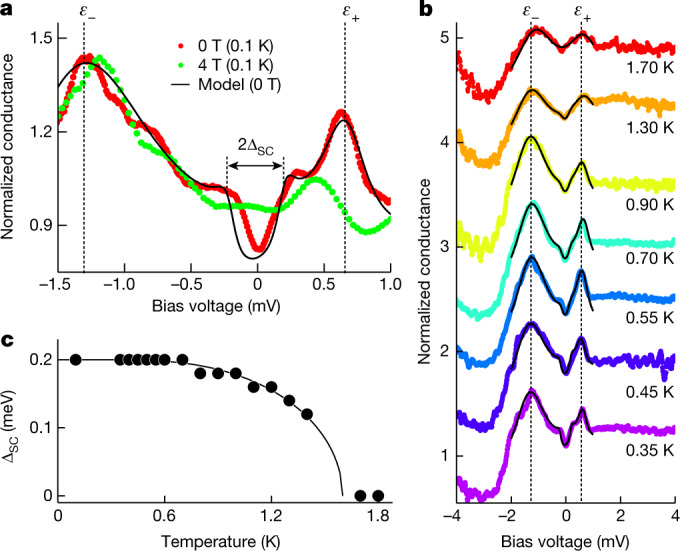
Superconductivity in the 2DHF. **a**, Tunnelling conductance versus bias voltage at zero field (red) and at 4 T (green). The fit at zero magnetic field is shown by the black line. The superconducting gap, Δ_SC_, is marked by an arrow. **b**, Tunnelling conductance versus temperature at zero field. The curves are shifted for clarity. The lines are fits to the model. **c**, Temperature dependence of the superconducting gap, Δ_SC_(*T*), is shown as black points. The BCS temperature dependence (line) is shown as a guide.

**Extended Data Fig. 8:**
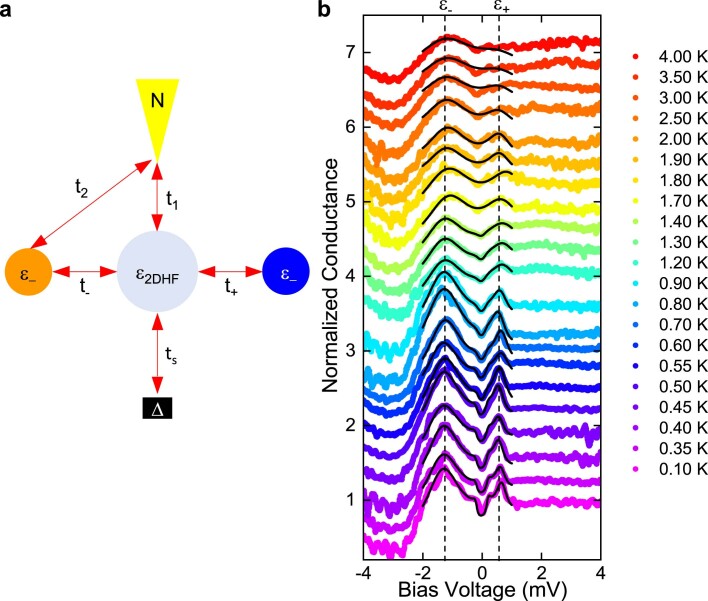
Model and tunnelling conductance from 0.1 K to 4 K. **a**, Schematic representation of the model and its parameters described in Methods. **b**, Tunnelling conductance as a function of temperature, also well above the superconducting transition temperature *T*_c_. Notice how the feature at *ε*_+_ vanishes because of thermal smearing, whereas the more pronounced feature at *ε*_−_ remains up to higher temperatures. Data are shown by coloured points and the black lines are fits, described in the text.

At the energy range below the superconducting gap, Δ_SC_ ≈ 200 μeV, we observe that there is a large zero-bias conductance (see Fig. 4a). There are indications for unconventional superconductivity in bulk URu_2_Si_2_, with a d-wave symmetry order parameter^29^. This can contribute to the suppression of the superconducting features in the tunnelling conductance, but it hardly leads to the zero-bias conductance observed in our experiment. Similar small-sized superconducting features are found in other heavy-fermion superconductors, such as CeCoIn_5_ or UTe_2_, and remain difficult to explain^36–38^. Most notably, macroscopic measurements such as specific heat or thermal conductivity provide, in all these systems, a negligible zero-temperature extrapolation of the electronic density, suggesting that the superconducting density of states at the Fermi level is very small^12^. The 2DHF is strongly coupled to the superconducting bulk states and the proximity effect from bulk superconductivity should provide only a small amount of states at low energies. However, it is important to consider the coupling of the 2DHF to strongly energy-dependent resonant states giving peaks in the tunnelling conductance as well. The concomitant broadening then leads to a large zero-bias tunnelling conductance. Using a model that takes this into account (see Methods and Extended Data Fig. 8), we can understand the main features of the tunnelling conductance and follow the superconducting gap with temperature (Fig. 4b,c). This solves the discrepancy between macroscopic and surface experiments and shows the relevance of two-dimensional electronic states to understanding the tunnelling conductance.

In summary, we have observed 2DHFs in terraced surfaces inside the HO phase of URu_2_Si_2_. The 2DHF exhibits quantum-well states with energy separation of fractions of a meV when confined between steps. The 2DHF is connected to the bulk heavy-fermion states. At steps, we observe a 1DES, which shows in-plane electronic symmetry breaking and inequivalent electronic arrangement in subsequent U layers in the HO phase. The discovery of 2DHFs and related confined states opens new possibilities to study the interplay of quantized heavy-fermion states and unconventional superconductivity, as several heavy-fermion materials show unconventional superconductivity in the bulk, often coexisting with other long-range ordered phases. Apart from URu_2_Si_2_, there are other heavy fermions, such as CeCoIn_5_, UBe_13_, UPt_3_ or UTe_2_, in which the proposed superconducting states are spin-singlet d-wave or spin-triplet p-wave and f-wave states. These could exhibit 2DHFs and the associated edge states could incorporate excitations with unique properties such as Majorana fermions following non-Abelian statistics. Furthermore, because the source of quantization is lateral confinement, correlated quantum-confined states can be obtained in nanostructures built on the surface by manipulation of adatoms or by controlling layer growth in thin films^15,16,39,40^. This opens new avenues to generate, isolate and manipulate excitations in unconventional superconductors.

## Methods

### STM experiments

Single crystals of URu_2_Si_2_ were grown by the Czochralski technique in a Tetra Arc Furnace. We scanned samples for a low residual resistivity and a high superconducting critical temperature, close to 1.5 K. Such samples were then cut in a bar shape with dimensions 4 × 1 × 1 mm^3^, with the long distance parallel to the *c* axis. We mounted the samples on the sample holder of a scanning tunnelling microscope. The scanning tunnelling microscope was mounted in a dilution refrigerator. The resolution in energy of the setup was tested by measuring the superconducting tunnelling conductance with the tip and sample of s-wave superconductors Al and Pb down to 100 mK (ref. ^41^). Details of image-rendering software are provided in refs. ^42,43^. The scanning tunnelling microscope head features a low-temperature movable sample holder, which is used to cleave the sample at cryogenic temperatures^41,44^. At the same time, and importantly for this study, the sample holder allows modifying many times the scanning window. The terraces discussed in this work were found in three different samples, after studying hundreds of fields of view.

### Surface termination in URu_2_Si_2_

We focus on U-terminated surfaces. In Extended Data Fig. 1a, we show the URu_2_Si_2_ crystal unit cell highlighting the U, Si and Ru planes; their inter-layer distances are indicated in units of the *c*-axis lattice parameter. In Extended Data Fig. 1b–d, we show STM images corresponding to different surface terminations. These surfaces are all obtained after cryogenic cleaving. On the surfaces full of square-shaped terraces, we find the results obtained in the main text. An example is shown in Extended Data Fig. 1b. All the observed terraces are separated by *c*/2 ≈ 4.84 Å. In Extended Data Fig. 1c,d, we show terraces with a triangular shape, in which we do not observe the phenomena discussed in the main text. Here the distance between consecutive terraces is about 0.11*c*, about 0.39*c* or about 0.61*c*, which correspond, respectively, to the three possible distances between U–Si planes (coloured arrows in Extended Data Fig. 1a). Therefore, we see that the surfaces with terraces having a triangular shape correspond to Si layers, sometimes with a U layer in between. By contrast, the surfaces with terraces having a square shape are U terminated. Atomically resolved images inside terraces (Extended Data Fig. 1e) provide the square atomic U lattice with an in-plane constant lattice of *a* = 4.12 Å . In Extended Data Fig. 1f, we show a typical atomic-sized image on Si-terminated surfaces. We do not observe atomic resolution and have sometimes seen circular defects. Defects in the U-terminated surfaces are very different, as shown in Extended Data Fig. 1g–o. We distinguish two distinct types of defect. The defects can be either point-like protrusions (Extended Data Fig. 1g,h) or troughs (Extended Data Fig. 1i). Sometimes, defects are arranged in small-sized square or rectangular structures (Extended Data Fig. 1j–o). Most of these defects are probably because of vacancies or interstitial atoms in layers below the U surface layer.

### Tunnelling conductance in the HO state

The tunnelling conductance of the HO state has been discussed in refs. ^45–49^. We have reproduced the results, as shown in Extended Data Fig. 2a,b. The tunnelling conductance results from simultaneous tunnelling into heavy and light bands, as in other heavy-fermion compounds^50–52^. The red line in Extended Data Fig. 2a for *T* = 18 K follows a Fano function

1 $$g(E)=A\frac{{\left(q+\left(E-{E}_{{\rm{Fano}}}\right)/{\Gamma }_{{\rm{F}}}\right)}^{2}}{\left(E-{E}_{{\rm{Fano}}}\right)/{\Gamma }_{{\rm{F}}}+1},$$

in which *A* is a constant of proportionality, *q* is the ratio between two tunnelling paths and *E*_Fano_ is the Fano resonance energy with width ${\Gamma }_{{\rm{F}}}=2\sqrt{{(\pi {k}_{{\rm{B}}}T)}^{2}+2{({k}_{{\rm{B}}}{T}_{{\rm{K}}})}^{2}}$, *T*_K_ being the Kondo temperature^45,46^. For the fit, we include an asymmetric linear background owing to the degree of particle–hole asymmetry in the light conduction band^45,53^. To account for the thermal broadening, we convolute the result with the derivative of the Fermi–Dirac distribution. We find *q* = 0.8 ± 0.5, *E*_Fano_ = 3 ± 1 mV, Γ_F_ = 22 ± 1 mV and *T*_K_ = 90 ± 5 K, consistent with previous reports^45,46^.

Inside the HO phase (red line in Extended Data Fig. 2b), we use the same Fano function, multiplied by an asymmetric BCS-like gap function with an offset *δ*_E_

2 $${g}_{{\rm{HO}}}=(E-{\delta }_{{\rm{E}}}-i{\gamma }_{{\rm{HO}}})/\left[[\sqrt{{(E-{\delta }_{{\rm{E}}}-i{\gamma }_{{\rm{HO}}})}^{2}-{\Delta }_{{\rm{HO}}}^{2}}]\right]$$

The resulting function is convoluted with the derivative of the Fermi–Dirac distribution function. We find *δ*_*E*_(4.1 K) = 1.5 ± 0.5 meV and Δ_HO_(4.1 K) = 4.0 ± 0.5 meV, consistent with previous reports^45,46^.

Note that we also observe further features at lower temperatures and smaller bias voltages (Extended Data Fig. 2c). The red line in Extended Data Fig. 2c is a fit described below. The features above the superconducting gap can also be roughly obtained by using two Lorentzians at *ε*_−_ and *ε*_+_ and an asymmetric background. Probably, the peaks at *ε*_−_ and *ε*_+_ are because of avoided crossings in the band structure of the 2DHF at very low energies. We notice that the small feature at *ε*_+_ occurs at a very similar energy range as a kink in the band structure found at the surface of Th-doped URu_2_Si_2_ (refs. ^48,49^). In the calculations we show below, we can identify features in surface f-derived bands that can be associated to such peaks in the tunnelling conductance. However, such features can form as a result of correlations elsewhere in the Brillouin zone as well.

### Quantum-well states at terraces between steps

In Extended Data Fig. 3a, we show the tunnelling conductance background subtracted from Fig. 2a to obtain Fig. 2b. To carry out the background subtraction, we first identify the features at *ε*_−_ and *ε*_+_ in the conductance map. These are the light-blue regions centred at *ε*_+_ and the red–yellow region centred at *ε*_−_ in Extended Data Fig. 3a. We then identify the edge states occurring at the steps, given by the red areas at the sides of Extended Data Fig. 3a. Similar peaks are obtained on steps separating different terraces. The nature and shape of the 1DESs is discussed below. We then model these features by a set of Lorentzians and obtain the pattern shown in Extended Data Fig. 3a. We subtract this pattern from the experiment (Fig. 2a) to obtain the pattern shown in Extended Data Fig. 3b.

To model the quantum-well states, we use the Fabry–Pérot interferometer expression for the density of states *g*_FP_(*x*, *E*) given by

3 $$\begin{array}{l}{g}_{{\rm{FP}}}(x,E)\propto {\int }_{0}^{k}\frac{{\rm{d}}q}{\sqrt{{k}^{2}-{q}^{2}}}\\ \times \frac{(1-{r}^{2})[1+{r}^{2}+2r\cos (2q(x-L)-\phi )]+(1-{r}^{2})[1+{r}^{2}+2r\cos (2qx+\phi )]}{1+{r}^{4}-2{r}^{2}\cos (2qL+2\phi )}\end{array}$$

with $k=\sqrt{2{m}^{* }(E-{E}_{0})/{\hbar }^{2}}$, m* the electronic effective mass, *r* the reflection amplitude, *ϕ* the phase and *L* the width of the terrace^23^. The Fabry–Pérot interferometer is an optical resonator made of semireflecting mirrors and provides a simple and insightful way to model electronic wavefunctions confined between two wells. More information on surface band structure and on quantum-well states by confinement is provided in refs. ^3,9,54–60^. We assume a symmetric potential well with *L* = 20 nm, *r* = 0.5 and *ϕ* = −π. The pattern generated by equation (3) is shown in Extended Data Fig. 3c. White points provide the positions of quantized levels as in Fig. 2a,b. It is not difficult to see that the structure of quantized levels is renormalized together with the electronic band structure. Smaller electronic effective masses imply larger quantized level width and separation, and vice versa. However, the simple Fabry–Pérot model does not take into account relaxation by electron–electron interactions, which lead to the extra level broadening discussed in the main text and in refs. ^27,28,61^.

The black lines in Fig. 2d are fits to the equation (3). To account for the behaviour at the edges, we add the equation (5) for the 1DES. We use the parameters extracted for the terrace *L*_3_, discussed in Extended Data Table 1. We show further examples in Extended Data Fig. 4. Note that, in Fig. 2d, we use equation (3) along with the contribution from the 1DES and contributions for *ε*_−_ and *ε*_+_ at the bias voltages at which these features are observed in the tunnelling conductance.

The 2DHF quantization was observed on the surfaces of different URu_2_Si_2_ samples. In Extended Data Fig. 5a–c, we show the result on another sample. Notice here that terraces have different sizes. We show in Extended Data Fig. 5a the STM topography image. In Extended Data Fig. 5b, we show a height profile through the white line in Extended Data Fig. 5a. In Extended Data Fig. 5c, we represent the tunnelling conductance along the central terrace (*L* ≈ 57 nm) of this profile. We observe similar tunnelling conductance curves as those presented in the main text. Notice the features at *ε*_−_ and *ε*_+_. The quantized levels are also readily observed. These occur, however, at different energy values, as the size of the terrace *L* is different to that of the terrace in the main text. In Extended Data Fig. 5d, we represent the values of the quantized levels found in terraces of different sizes *L* by different colours; we show the dispersion relation of the 2DHF as a magenta line.

In Extended Data Fig. 5e, we show as coloured points the bias voltage dependence of the energy spacing Δ*E* between consecutive quantized levels for terraces *L*_3_, *L*_4_, the terrace with length *L* = 57 nm (shown in Extended Data Fig. 5c) and a terrace with length *L* = 27 nm (not shown). We can write that $\Delta E={E}_{n+1}-{E}_{n}=\left(\frac{{\hbar }^{2}{\pi }^{2}}{2{m}^{* }{L}^{2}}\right)\left({(n+1)}^{2}-{n}^{2}\right)$, with *n* = 1, 2, 3,…. This gives a square-root dependence of Δ*E* on the energy, $\Delta E={E}_{n+1}-{E}_{n}=\left(\frac{{\hbar }^{2}{\pi }^{2}}{2{m}^{* }{L}^{2}}\right)\left(2n+1\right)\propto \sqrt{E}$, shown in Extended Data Fig. 5e. In Extended Data Fig. 5f, we plot the average value of $\frac{\Delta E}{2n+1}$ for each terrace as a function of *L*. We find the expected $\frac{1}{{L}^{2}}$ dependence.

Note that in Extended Data Fig. 5 and Fig. 1d, we do not perform any symmetrization. We can see a tendency of the quantized states to shift towards the sides of the terrace, giving intensity patterns that are slightly asymmetric. We have calculated the expected patterns for different reflection coefficients at each side of the terrace. This produces asymmetric patterns similar to those observed experimentally. However, it is difficult to separate such an asymmetry from the signal coming from the edge states in the tunnelling conductance. Lateral symmetrization thus remains the best way to analyse and understand quantum states in the terraces observed here in URu_2_Si_2_.

We use $\hbar /\tau ={\Gamma }_{0}+\left(| {E}_{0}| /4\pi \right){\left[(E+| {E}_{0}| )/{E}_{0}\right]}^{2}$ to fit the energy dependence of the lifetime^61^. The parameter |*E*_0_|/4π is a prefactor that fits our experiment well. The prefactor is sometimes provided as a number^28^ and has also been estimated as $\frac{{e}^{2}{k}_{{\rm{F}}}^{2}\pi }{4\pi {{\epsilon }}_{0}\,32\,\hbar {q}_{{\rm{TF}}}}$ or, equivalently, $\frac{e{m}^{3/2}}{32\times {3}^{5/6}{\pi }^{2/3}{{\epsilon }}_{0}^{1/2}{\hbar }^{4}{n}^{5/6}}$ (with *ϵ*_0_ the dielectric constant, *n* the electron density and *q*_TF_ the Thomas–Fermi screening length)^27^. These estimations provide similar values to |*E*_0_|/4π.

To obtain the energy dependence of the reflection coefficient, *r*(*E*), we used the model described in ref. ^24^. To this end, we consider a one-dimensional periodic array of scattering objects, each modelled by a square potential well of width *b* < *L* (*L* is the width of the terrace). A constant complex potential *W* provides confinement and coupling to the bulk states. We can then write

4 $$R(E)=\frac{{e}^{iqb}-{e}^{-iqb}}{{e}^{iqb}\left(\frac{k-q}{k+q}\right)-{e}^{-iqb}\left(\frac{k+q}{k-q}\right)}{e}^{-ikb}$$

with $k=\sqrt{\frac{2{m}^{* }E}{{\hbar }^{2}}}$ and $q=\sqrt{\frac{2{m}^{* }(E-W)}{{\hbar }^{2}}}$. The reflection coefficient *r*(*E*) is given by *r*(*E*) = |*R*(*E*)|^2^. For the dashed line in Fig. 2e, we use typical parameters for Cu, with m* = 0.46*m*_0_ and *W* = (−2 − 1*i*) eV. We shift the obtained curve in energy to obtain a result within the energy range of our data. For the continuous line in Fig. 2e, we use m* = 17*m*_0_ and *W* = (−18 − 5*i*) meV.

### Band-structure calculations at U-terminated surfaces of URu_2_Si_2_

The band structure of bulk URu_2_Si_2_ has been analysed previously in detail using DFT calculations^62–64^. Relevant results coincide with angle-resolved photoemission, STM and quantum oscillation studies^22,65–71^.

Several surface states have been observed by angle-resolved photoemission spectroscopy^19–22^. The surface state discussed in refs. ^19,20^ is formed by a hole-like band with its maximum at −35 meV and is thus far from what we observe here. At the *X* point of the Brillouin zone, there are no bulk states. Angle-resolved photoemission spectroscopy measurements show hints of surface-like bands with two-dimensional character at these points^21^. We have taken a closer look at the *X* point through DFT calculations. To this end, we built a U-terminated supercell consisting of 37 atomic layers, giving a total of ten U layers (Extended Data Fig. 6a). We performed DFT calculations using the full-potential linearized augmented plane-wave method with local orbitals as implemented in the WIEN2k package^72^. Atomic spheres radii were set to 2.5, 2.5 and 1.9 Bohr radii for U, Ru and Si, respectively. We used a 19 × 19 × 1 mesh of *k*-points in the first Brillouin zone, reduced by symmetry to 55 distinct *k*-points. The RK_max_ parameter was set to 6.5, resulting in a basis size of approximately 5,400 (more than 100 basis functions per atom). Spin–orbital coupling was included in the second variational step^73^ and relativistic local orbitals were included for U 6*p*_1/2_ and Ru 4*p*_1/2_ states. The basis for calculations of the spin–orbital eigenvalue problem consisted of scalar-relativistic valence states of energies up to about 5 Ry, resulting in a basis size of about 3,800. The local density approximation was used for the treatment of exchange and correlation effects^63,74^.

In Extended Data Fig. 6b, we highlight in particular the U spin-up character of the obtained surface-projected band structure. The spin-down character is much less pronounced within the shown energy range. There are several bands inside gaps of the bulk band structure, but only those around the *X* point of the simple tetragonal Brillouin zone, *X*_st_ (see Extended Data Fig. 6c), are sufficiently shallow to provide large effective masses.

We find a surface state (upper inset of Extended Data Fig. 6b) that consists of two hybridized hole bands, forming an M-shaped feature close to the Fermi level. The dispersion relation found in our experiment (magenta line in the upper inset of Extended Data Fig. 6b) is compatible with the central part of the M-shaped feature.

### 1DES and HO within U layers

To analyse the 1DES at the step between two terraces, we use a one-dimensional Dirac-function-like potential at the step, *V*(*x*) = *U*_0_*δ*(*x* − *x*_1DES_), in which *x*_1DES_ is the position of the 1DES. We take *U*_0_ = *b*_0_*V*_0_, with *b*_0_ the width of the potential well and *V*_0_ the energy depth (*V*_0_ < 0). We add a complex potential, *V*(*x*) → (*U*_0_ − *iU*_1_)*δ*(*x* − *x*_1DES_) to simulate the coupling of the 1DES to the bulk of the crystal. A schematic representation of this model is shown in Extended Data Fig. 7a. Solving the Schrödinger equation for *E* < 0, we obtain the Green’s function of the states in the potential well

5 $$G(E)=A\frac{{e}^{-| x-{x}_{{\rm{1DES}}}| /{\lambda }_{x}}}{E-{E}_{{\rm{1DES}}}+i{\eta }_{{\rm{1DES}}}},$$

in which ${\lambda }_{x}=\frac{{\hbar }^{2}}{{m}^{* }}| {U}_{0}{| }^{-1}$ is the decay length with m* the effective mass. *E*_1DES_ and *η*_1DES_ are the energy position and the energy broadening of the 1DES given by

6 $${E}_{{\rm{1DES}}}=\delta V+{E}_{1}=\delta V-\frac{{m}^{* }}{{\hbar }^{2}}\left({U}_{0}^{2}-{U}_{1}^{2}\right)$$

7 $${\eta }_{{\rm{1DES}}}=-\frac{{m}^{* }}{{\hbar }^{2}}{U}_{0}{U}_{1}$$

in which *δV* is the height of the potential barrier of the well relative to the Fermi level.

We can now fit the tunnelling conductance at the 1DES using

8 $${g}_{{\rm{1DES}}}={A}_{0}\frac{{\eta }_{{\rm{1DES}}}{e}^{-\frac{\left|x-{x}_{{\rm{1DES}}}\right|}{{\lambda }_{x}}}}{{\left(E-{E}_{{\rm{1DES}}}\right)}^{2}+{\eta }_{{\rm{1DES}}}^{2}}$$

convoluted with the derivative of the Fermi–Dirac distribution function. Extended Data Table 1 shows the extracted fitting parameters *E*_1DES_, *η*_1DES_, *λ*_*x*_ and *x*_1DES_ for the four different terraces *L*_1_ to *L*_4_ from Fig. 1.

From Extended Data Table 1, we see that the energy position and the energy broadening of the 1DES are independent of the terrace size, with average values of *E*_1DES_ = −0.52 ± 0.14 meV and *η*_1DES_ = 0.45 ± 0.06 meV. We also see that all the spatial features are always at the same position with respect to the step, *x*_1DES_ ≈ 4.0*a*_0_, *a*_0_ being the in-plane lattice constant, with a decay length *λ*_*x*_ ≈ 0.9 nm ≈ 2*a*_0_. The latter indicates that 1DESs and 2DHFs couple when the decay length reaches a few interatomic distances. With the extracted average values from Extended Data Table 1 for *λ*_*x*_, *η*_1DES_ and *E*_1DES_, we obtain *U*_0_ = 5.4 meVÅ, *U*_1_ = 0.38 meVÅ and *δV* = 3.1 meV.

We can analyse the 1DES through the tunnelling conductance at a step (Extended Data Fig. 7b,c). At low bias voltages, we find a dip in the tunnelling conductance of a few nanometres at the upper side of the step (blue lines in Extended Data Fig. 7b; for example, at −1.2 mV). The dip fills with the 1DES at about *E*_1DES_ (red lines in Extended Data Fig. 7b at −0.4 mV) and empties again at higher bias voltages. This shows that charge depletion close to the step opens a gap in the band structure. The gap is filled at the resonant energy of the 1DES, as observed previously in metals^30,75,76^. By normalizing the tunnelling conductance to its shape far from the step (Extended Data Fig. 7c), we can follow the decay of the 1DES into the quantum-well states of the 2DHF with the model described above (Extended Data Fig. 7a). The decay length is on the order of the inverse of the wavevector of the 2DHF.

Taking a closer look at the steps, we surprisingly find a notable in-plane anisotropy of the 1DES. As we see in Extended Data Fig. 7b, the 1DES is observed when crossing steps along the dashed red line in Extended Data Fig. 7b but not along the dashed blue line, as discussed in the main text. It is useful here to take a closer look at the HO as well. As discussed in refs. ^14,34,64,77–79^, there is no dipolar (magnetic) or structural order related to the HO phase. Instead, the U lattice can present some sort of long-range electronic ordering, whose actual symmetry and shape is considered as a relevant and open mystery^14^.

Previous NMR measurements indicated the absence of fourfold in-plane symmetry breaking in bulk URu_2_Si_2_ (ref. ^35^), whereas our STM data clearly show an in-plane symmetry breaking in the 1DES. At the surface, there can be marked changes in the electronic structure owing to a modification of the valence of uranium atoms^21,80^. Rather, the breaking of the in-plane symmetry observed here in the 1DES suggests that a fundamental breaking of the near-surface electronic properties of the U lattice is at play in the HO phase.

### Interplay between superconductivity and the 2DHF

We consider several parallel conduction channels between the tip and the surface. For simplicity, we take into account tunnelling into the 2DHF and into the feature of largest size at *ε*_−_ (Extended Data Fig. 8a). The first channel, *t*_1_, connects the tip with the 2DHF. The 2DHF is superconducting by proximity from the bulk superconductor, which we model using a coupling *t*_s_. With the second channel, *t*_2_, we connect the tip to other surface features. We can write the retarded Green function ${\hat{G}}^{{\rm{r}}}$ as

9 $${\hat{G}}^{{\rm{r}}}={\left(\begin{array}{cc}{\hat{M}}_{{\rm{2D}}} & {\hat{t}}_{-}\\ {\hat{t}}_{-} & {\hat{M}}_{-}\end{array}\right)}^{-1}$$

with

10 $$\begin{array}{l}{\hat{M}}_{{\rm{2D}}}\,=\,\left(\begin{array}{cc}E-{E}_{{\rm{2DHF}}}-\frac{{t}_{+}^{2}}{E-{E}_{+}}+\frac{E+i\eta }{\Omega }{\bar{t}}_{{\rm{s}}} & \frac{\Delta }{\Omega }{\bar{t}}_{{\rm{s}}}\\ \frac{\Delta }{\Omega }{\bar{t}}_{{\rm{s}}} & E+{E}_{{\rm{2DHF}}}^{* }-\frac{{t}_{+}^{2}}{E+{E}_{+}^{* }}+\frac{E+i\eta }{\Omega }{\bar{t}}_{{\rm{s}}}\end{array}\right)\\ \,\,{\hat{M}}_{-}\,=\,\left(\begin{array}{cc}E-{E}_{-} & 0\\ 0 & E+{E}_{-}^{* }\end{array}\right)\\ \,{\hat{t}}_{-}\,=\,\left(\begin{array}{cc}{t}_{-} & 0\\ 0 & -{t}_{-}\end{array}\right)\\ \,{E}_{j}\,=\,{\varepsilon }_{j}-i\frac{{\Gamma }_{j}}{2}\,(j={\rm{2DHF}},-,+)\\ \,{\bar{t}}_{{\rm{s}}}\,=\,\frac{{t}_{{\rm{s}}}^{2}}{W}\\ \,\Omega \,=\,\sqrt{{\Delta }^{2}-{(E+i\eta )}^{2}}\end{array}$$

in which *E*_2DHF_ is the energy associated to the 2DHF and includes the shift of energy owing to HO and Fano resonance, *W* is an energy scale related to the normal density of states at the Fermi level by *ρ*(*E*_F_) = 1/(π*W*), Δ is the superconducting gap and *η* is a small energy relaxation rate. We have added the self energies *i*Γ_*j*_/2, (*j* = 2DHF, −, +), with Γ_*j*_ the width of the resonance *j*.

The differential conductance is calculated as

11 $$\sigma (V)={\sigma }_{0}\int T(E)\left(-\frac{{\rm{d}}f(E-eV,T)}{{\rm{d}}\left(eV\right)}\right){\rm{d}}E$$

with

12 $$\begin{array}{l}T(E)=-\frac{1}{\pi }{\rm{Im}}({G}_{11}^{{\rm{r}}}(E){t}_{1}^{2}+{G}_{33}^{{\rm{r}}}(E){t}_{2}^{2}\\ \,\,+{t}_{1}{t}_{2}({G}_{13}^{{\rm{r}}}(E)+{G}_{31}^{{\rm{r}}}(E)))\end{array}$$

Here *f*(*E*, *T*) is the Fermi–Dirac distribution at the energy *E* and temperature *T* and ${\sigma }_{0}=\frac{2{e}^{2}}{h}$ is the quantum of conductance (with *h* being Planck’s constant and *e* the elementary charge). Notice that *T* is the transmission, not the density of states often used to discuss STM measurements in superconductors. Notice also that we take into account tunnelling into the 2DHF (${G}_{11}^{{\rm{r}}}$) and into *ε*_−_ (${G}_{33}^{{\rm{r}}}$), with mixed contributions (${G}_{31}^{{\rm{r}}}$ and ${G}_{13}^{{\rm{r}}}$).

To fit the tunnelling conductance curves shown in Fig. 4a,b and Extended Data Fig. 8b, we subtracted an asymmetric background (see Extended Data Figs. 2 and 8b). In Extended Data Table 2, we show the parameters used to obtain the tunnelling conductance with temperature (shown as black lines in Fig. 4a,b) from equation (11). We do not vary the parameters *η* = 0.018 meV, *ε*_2DHF_ = 12 meV, Γ_2DHF_ = 1 meV, Γ_−_ = 0.55 meV and Γ_+_ = 0.14 meV with temperature. We see that the superconducting lifetime itself is practically negligible, *η* = 0.018 meV ≪  Δ = 0.2 meV. The large zero-bias conductance is not because of the incomplete coupling to the bulk, as *t*_s_ is close to one. There is further smearing coming from features at *ε*_+_ and *ε*_−_. Γ_+_ and Γ_−_ provide the smeared superconducting density of states and a finite tunnelling conductance at zero bias. Notice that the superconducting gap vanishes at the critical temperature *T*_c_ but that the strongly bias-voltage-dependent tunnelling conductance remains up to higher temperatures (Extended Data Fig. 8).

There are numerous evidences for d-wave or more complex superconducting properties in URu_2_Si_2_. The differences in the superconducting density of states between these superconducting states and s-wave superconductivity are relatively small, particularly because the tunnelling conductance obtained with the model described here provides smeared conductance curves. The same occurs for the temperature dependence of the superconducting gap. Instead, the shape and asymmetry of the tunnelling conductance at defects might provide the connection between the properties of the superconducting 2DHF and the unconventional superconductivity of the bulk.

### Results at point defects

**Extended Data Fig. 9:**
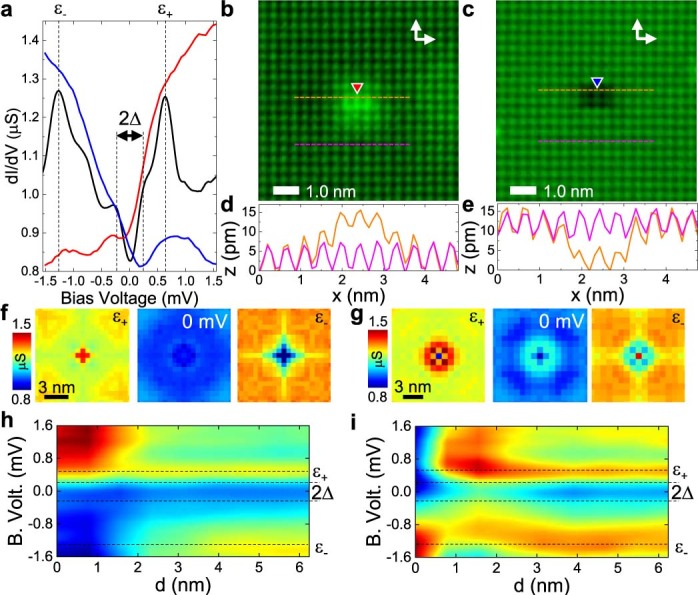
Tunnelling conductance at atomic-sized defects. **a**, Tunnelling conductance obtained far from defects (black line). We show by the vertical dashed lines the features at *ε*_+_ and *ε*_−_. Curves are taken at 0.1 K. **b**,**c**, Topographic images of the U lattice showing two types of defect. White arrows provide the in-plane lattice constants. The white scale bar is also shown. The red and blue triangles provide the positions at which the red and blue curves in **a** were taken. **d**,**e**, Profiles obtained along the dashed lines of **b** and **c** with the same colours. **f**,**g**, Tunnelling conductance maps obtained at the bias voltages shown in each panel, using the colour scale provided on the left for protrusions and troughs, respectively. The scale bar is given in black. To increase the signal-to-noise ratio, we have made a fourfold symmetrization in all these panels. **h**,**i**, Conductance across the defect site as a function of the distance from the centre of the defects in **b** and **c**, respectively, along a crystalline direction. The colour scale is given by the bar on the left of **f** and **g**.

We analyse in more detail here the tunnelling conductance at defects. We plot the tunnelling conductance obtained on two different defects in Extended Data Fig. 9a as blue and red lines. Notice the pronounced electron–hole asymmetry, which provides curves that widely differ from the curves far from defects (black line in Extended Data Fig. 9a). As discussed above, we can identify two kinds of defects: protrusions with height increases of around 15 pm, probably because of interstitial atoms located beneath the surface (Extended Data Fig. 9b,d,f,h), and troughs of around 15 pm in size, probably because of vacancies beneath the surface (Extended Data Fig. 9c,e,g,i). The defects visibly affect the tunnelling conductance. We plot the tunnelling conductance at *ε*_+_, 0 mV and *ε*_−_ for both types of defect in Extended Data Fig. 9f,g. In Extended Data Fig. 9h,i, we show the spatial dependence of the tunnelling conductance along a crystalline axis for both types of defect. At the site of the defect, there is a pronounced electron–hole asymmetry, which is opposite for each kind of defect. Protrusions provide a substantially enhanced tunnelling conductance for empty states above the Fermi level, whereas troughs provide the opposite (Extended Data Fig. 9a). At zero bias, the troughs show a pronounced in-plane anisotropy and a reduction of the superconducting gap, whereas the protrusions are in-plane isotropic and show an opened superconducting gap. When leaving the defect, the usual behaviour is recovered after several nanometres (Extended Data Fig. 9h,i).

To explain the pronounced modification of the electron–hole asymmetry of the tunnelling conductance, let us consider electron correlations creating a large Fermi surface scenario at some portion of the band structure. At low temperatures, correlations provide an avoided band crossing owing to hybridized heavy and light bands. We can assume that it happens somewhere in the band structure of URu_2_Si_2_, for example, close to the Γ point, as suggested in refs. ^14,18,47^. Then, we obtain at low temperatures a large Fermi surface with heavy electrons and a close-lying light band with smaller wavevectors above the Fermi level. We can then assume that both kinds of defect couple to different parts of such a correlated band structure. Following our experiments, protrusions create 2DHF coupling to the small light band and a large density of states for empty states above the Fermi level (red curve in Extended Data Fig. 9a). Troughs can instead favour coupling to the heavy band, giving the opposite behaviour (blue curve in Extended Data Fig. 9a). The superconducting gap is disturbed at troughs. Generally, we expect all sorts of defects to be pair breaking, as URu_2_Si_2_ is not a s-wave superconductor. However, two-dimensional quantized states screen pair-breaking interactions from the underlying superconducting bulk^81^. This suggests that the absence of in-gap states in protrusions is because of screening by the 2DHF. Troughs, however, produce a strong coupling to the heavy bands that carry the heavy superconducting state and thus also contribute to pair breaking.

In-gap states at troughs have a certain in-plane anisotropy (Extended Data Fig. 9g, middle panel). The anisotropy of the superconducting gap has been analysed with macroscopic measurements^82–85^. There are indications for gap nodes, for instance, from specific heat measurements^82,83,85^. Local vortex dynamics shows an in-plane fourfold or twofold pinning potential^86^. When measured as a function of the angle, there is a pronounced out-of-plane anisotropy, which has been associated to nodes along the *c* axis, but there is little in-plane variation^85^. Following such a nodal structure, several experiments propose a chiral *k*_*z*_(*k*_*x*_ + *ik*_*y*_) superconducting wavefunction^84,85,87^. The surface states of a (*k*_*x*_ ± *ik*_*y*_) superconductor are predicted to be chiral arc states connecting the Weyl nodes^88^. It is so far unclear how these surface states of the superconducting phase adapt to the surface states of the normal phase, which appear as a consequence of the surface-induced perturbation of the crystalline potential. As mentioned, we observe an asymmetry at zero bias (Extended Data Fig. 9g, middle panel). To increase the signal-to-noise ratio, we were forced to apply a fourfold symmetrization. Thus, the anisotropy might be twofold or fourfold. The chiral *k*_*z*_(*k*_*x*_ + *ik*_*y*_) state is in-plane isotropic. However, the shape of in-gap states is determined by the anisotropy of the normal-state wavefunctions, together with the symmetry of the superconducting wavefunction, so that the observed in-plane anisotropy of the in-gap states probably reflects the normal-state in-plane anisotropy.

## Online content

Any methods, additional references, Nature Portfolio reporting summaries, source data, extended data, supplementary information, acknowledgements, peer review information; details of author contributions and competing interests; and statements of data and code availability are available at 10.1038/s41586-023-05830-1.

## Figures and Tables

**Extended Data Table 1:**
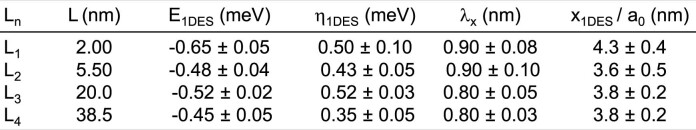
1DES parameters for terraces *L*_1_ to *L*_4_

Values of the parameters extracted from equation (8) used to fit the 1DES on the four different terraces *L*_1_ to *L*_4_ of Fig. 1.

**Extended Data Table 2:**
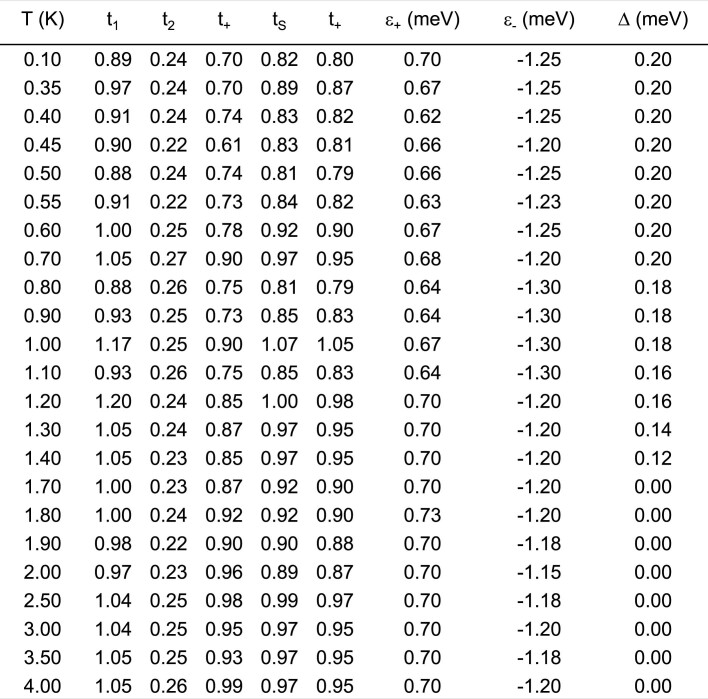
Fitting parameters from the tunnelling conductance in the range 0.1 K ≤ *T* ≤ 4.0 K

Values of the parameters extracted from equation (11) used to fit the tunnelling conductance curves shown as black lines in Fig. 4a,b and Extended Data Fig. 8b.

## Data Availability

All data supporting the findings of this study are available from the corresponding authors on request.

## Code availability

The simulation code WIEN2k used to compute the surface electronic band structure can be obtained from http://susi.theochem.tuwien.ac.at.
